# Veterinarians’ perspectives on animal welfare, legal enforcement, and forensic readiness in companion animal cruelty investigations in Thailand: A cross-sectional qualitative study

**DOI:** 10.14202/vetworld.2026.725-744

**Published:** 2026-02-26

**Authors:** Athip Lorsirigool, Yuttana Sudjaroen, Narong Kulnides, Natapol Pumipuntu, Atthaporn Roongsitthichai, Supawadee Piratae, Manakant Intrakamhaeng, Surangkanang Chaiyasak, Kanokpon Saenkaew

**Affiliations:** 1 Department of Forensic Science, Graduate School, Suan Sunandha Rajabhat University, Dusit District, 10300, Bangkok, Thailand; 2 Faculty of Science and Technology, Suan Sunandha Rajabhat University, Dusit District, 10300, Bangkok, Thailand; 3 One Health Research Unit, Mahasarakham University, 44000, Maha Sarakham, Thailand; 4 Faculty of Veterinary Sciences, Mahasarakham University, 44000, Maha Sarakham, Thailand; 5 Department of Laboratory Medicine, Pathology and Medical Genetics, Vancouver Island Health Authority, British Columbia, Canada; 6 Veterinary Public Health, Faculty of Veterinary Sciences, Mahasarakham University, 44000, Maha Sarakham, Thailand; 7 Veterinary Infectious Diseases Research Unit, Faculty of Veterinary Sciences, Mahasarakham University, 44000, Maha Sarakham, Thailand; 8 Faculty of Forensic Science, Royal Police Cadet Academy, 73110, Nakhon Pathom, Thailand

**Keywords:** animal cruelty, animal welfare law, forensic veterinary science, qualitative analysis, Thailand, veterinarian attitudes, veterinary forensics, welfare enforcement

## Abstract

**Background and Aim:**

Animal cruelty is a persistent concern for animal welfare and society worldwide. In Thailand, the Prevention of Cruelty to Animals and Animal Welfare Act B.E. 2557 (2014) provides a legal framework to address animal abuse; however, effective enforcement remains constrained by limitations in investigative procedures and the limited integration of veterinary forensic science. Veterinarians from the Department of Livestock Development (DLD) play a central role in responding to cruelty reports, conducting inspections, and supporting legal processes. This study aimed to explore the attitudes of DLD veterinarians toward companion animal welfare, the effectiveness of existing animal welfare legislation, and the current and potential role of forensic science in animal cruelty investigations in Thailand.

**Materials and Methods:**

A cross-sectional qualitative study was conducted between August 2024 and December 2024. Eighteen veterinarians with DLD from six geographic regions of Thailand were recruited using stratified purposive sampling. Data were collected via a validated, open-ended online questionnaire that addressed animal welfare challenges, legal enforcement, investigative procedures, and forensic applications. Qualitative content analysis was performed using the NVivo software, with double-blind coding to enhance analytical rigor. The codes were organized into categories and overarching themes following inductive thematic analysis.

**Results:**

Multiple interrelated drivers of companion animal cruelty were identified by veterinarians. The major welfare-related themes included a lack of owner responsibility and preparedness, insufficient knowledge and ethical awareness regarding animal care, socioeconomic constraints, deficiencies in stray animal management systems, weaknesses in law enforcement, and cultural attitudes that undermine animal welfare. While most respondents perceived the existing animal cruelty law as partially effective in reducing abuse, they highlighted critical gaps, including ambiguous legal definitions, inconsistent enforcement, and limited public awareness. The absence of standardized veterinary forensic protocols, limited forensic training, and restricted inspection authority were the primary factors driving investigative challenges. Respondents strongly supported the integration of forensic science, emphasizing its potential to improve evidentiary reliability, strengthen legal credibility, and enhance investigative outcomes, while also identifying the need for structured training programs and interagency collaboration.

**Conclusion:**

Companion animal cruelty in Thailand is driven by multifactorial welfare, legal, and societal challenges. Although current legislation provides a foundation for protection, the absence of standardized forensic practices limits investigative effectiveness. Strengthening veterinary forensic capacity through harmonized protocols, targeted training, and coordinated enforcement could substantially enhance animal welfare protection and support consistent application of animal cruelty legislation within a One Welfare framework.

## INTRODUCTION

Animal welfare is a global concern that emphasizes ensuring animals achieve an appropriate physical and mental quality of life in accordance with the fundamental needs of each species [1]. The assessment of animal welfare has traditionally been guided by the Five Freedoms principle, which encompasses freedom from hunger and thirst, discomfort, pain, restriction of natural behavior, and fear or distress. This framework was subsequently expanded into the Five Domains model of animal welfare assessment, comprising nutrition, environment, health, behavior, and mental state, thereby providing a more comprehensive and structured approach to welfare evaluation [2]. In parallel, the One Welfare concept highlights the interconnectedness of animal welfare, human well-being, and the environment, acknowledging that positive changes in one domain can generate beneficial effects across the others [3]. Consequently, many countries have introduced legal frameworks to prevent animal cruelty, and failure to provide appropriate animal welfare may expose animal owners to legal liability [4].

Most countries in Asia, Europe, and the Americas have enacted legislation to prevent cruelty toward companion animals, although the content and enforcement of these laws vary by local values, traditions, and cultural contexts [5]. These legal instruments primarily seek to establish and maintain acceptable standards of animal welfare, with noncompliance potentially resulting in penalties such as imprisonment or monetary fines [6]. In Thailand, the Prevention of Cruelty to Animals and the Welfare of Animals Act B.E. 2557 (2014) stipulates in Section 20 that acts of cruelty against animals constitute criminal offenses punishable by imprisonment for up to two years, a fine of up to approximately USD 1,200, or both. Furthermore, failure to provide appropriate animal welfare as outlined in Sections 22, 23, and 24 may result in a fine not exceeding approximately USD 1,200 [7]. Despite the presence of such legislation, the investigation of animal cruelty cases and situations involving inadequate companion animal welfare remains challenging for responsible authorities. The acquisition of reliable and objective evidence is therefore critical for effective legal adjudication, and the principles of forensic science are increasingly being applied to support and strengthen these investigative processes [8, 9].

Veterinary forensics refers to the application of scientific principles to the systematic analysis, documentation, and handling of evidence in cases involving animals, including companion animal abandonment, non-accidental injuries, illegal killings, and the trafficking of carcasses or body parts from protected or endangered wildlife species [10]. In recent years, veterinary forensics has been increasingly utilized in the investigation of unnatural deaths among companion animals [11]. Investigations at scenes involving companion animals should adhere to the same chain-of-custody principles applied in human forensic investigations, incorporating careful examination of the surrounding environment, the animal, and any potential weapons or objects associated with the incident [12]. Specialized diagnostic and investigative techniques, such as radiology, toxicology, histopathology, and necropsy, are commonly employed to establish the cause and manner of death, particularly in cases where animal cruelty is suspected [13].

Despite comprehensive animal welfare legislation and the growing recognition of forensic science as a valuable tool in animal cruelty investigations, significant gaps remain in the practical implementation and integration of these frameworks. In many countries, including Thailand, animal welfare laws primarily emphasize legal provisions and penalties, while giving limited attention to operational challenges encountered during investigations, such as evidence collection, documentation, and forensic interpretation. Empirical data on how frontline veterinarians perceive animal welfare enforcement, legal adequacy, and investigative readiness are scarce, particularly in relation to the systematic application of forensic science in companion animal cruelty cases. Moreover, there is a lack of nationally representative qualitative evidence examining whether current investigative practices align with established forensic principles and how these limitations may affect legal outcomes. The absence of standardized veterinary forensic protocols and structured training further exacerbates inconsistencies in case handling, highlighting the need for context-specific research that bridges animal welfare, law enforcement, and forensic practice.

The aim of this study was to explore veterinarians’ perspectives on companion animal welfare, the effectiveness of existing animal welfare legislation, and the current and potential role of forensic science in animal cruelty investigations in Thailand. Specifically, the study sought to identify the perceived drivers of companion animal cruelty, assess the challenges associated with legal enforcement and investigative procedures, and examine veterinarians’ views on integrating forensic science into routine animal welfare investigations.

## MATERIALS AND METHODS

### Ethical approval

Prior to the commencement of the study, ethical considerations related to both human participants and animals were addressed. The research involved only licensed veterinarians and did not include any direct interaction with animals; therefore, animal ethics approval was not required, and an exemption was granted by the institutional ethics committee.

The study protocol, including recruitment procedures, participant information sheets, consent format, questionnaire structure, confidentiality safeguards, and data management procedures, was reviewed and approved by the Human Research Ethics Committee of Suan Sunandha Rajabhat University, Thailand (Approval No. COA.2-008/2025). This approval confirmed compliance with the Ethics Guidelines of the National Research Council of Thailand, the Declaration of Helsinki, and Thailand’s Personal Data Protection Act (PDPA B.E. 2562), which govern the collection and processing of personal information in digital environments.

Participation was entirely voluntary. Before accessing the questionnaire, participants were provided with an electronic information sheet outlining the study objectives, procedures, expected duration, potential benefits, and the absence of risks or incentives. Informed consent was obtained electronically by requiring participants to select the “Agree to Participate” option before beginning the questionnaire. No identifying information, including names, addresses, telephone numbers, or employee identification numbers, was collected. To ensure anonymity, demographic information was recorded only in aggregated categories. The informed consent materials were provided in Thai, and the English version was translated using Google Translate and Grammarly.

### Study period and location

The cross-sectional study was conducted between August 1 and December 31, 2024. The study sites were purposively selected to represent the six regions of Thailand.

### Study design

This study employed a qualitative research design. The questionnaire was structured into two main sections. The first section collected general demographic information, including gender, age, and length of employment within the organization. The second section consisted of open-ended questions designed to elicit in-depth insights into respondents’ perspectives on animal cruelty and the application of forensic science in veterinary contexts.

The open-ended questions addressed four core areas. First, respondents were asked to describe their views on animal cruelty and inadequate animal welfare, including perceived underlying causes. Second, they were asked to evaluate the effectiveness of the Prevention of Cruelty to Animals and Welfare Act B.E. 2557 (2014) in mitigating pet abuse and to suggest potential legislative improvements. Third, respondents were asked to share their perceptions of procedures used in investigating reported animal cruelty cases, particularly whether these procedures align with standard forensic practices applied in human investigations and which protocols are followed within their respective organizations. Fourth, participants were asked to express their views on the potential benefits of applying FS methods in animal cruelty investigations, as well as their opinions on the value of training programs or courses related to the application of FS principles in veterinary practice.

Before data collection, the questionnaire was reviewed by three subject-matter experts in veterinary medicine, law, and forensic science to assess content validity using the item–objective congruence (IOC) method. All items achieved an IOC score of 1.00, indicating full agreement among experts regarding clarity, relevance, and alignment with the study objectives.

### Population and sample size

**Figure 1:**
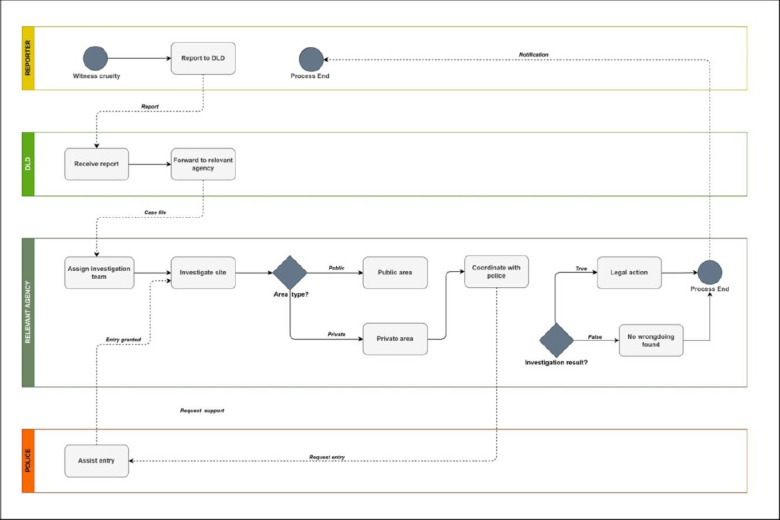
Reporting pathway for pet cruelty cases within the Department of Livestock Development (DLD), Thailand. When pet cruelty is observed, the incident is reported to the DLD via telephone or a mobile application. The information is forwarded to the relevant animal welfare division, which deploys a team for investigation. If the incident occurs in a public area and wrongdoing is confirmed, legal action is initiated. Access to private property requires coordination with the police or other authorized personnel. Following the investigation, a report is prepared indicating whether a criminal offense has been established (figure developed based on guidance from Nontachai Santichat and Arunroj Kullaya, DLD).

The study population consisted of veterinarians in Thailand. The sample comprised veterinarians employed by the Department of Livestock Development (DLD), the agency responsible for receiving reports of animal cruelty and enforcing animal welfare legislation in Thailand (Figure 1). The DLD field units operate in nearly all provinces across the country. The sampling frame was based on the official DLD staff directory, which lists all full-time veterinarians working at provincial and district levels.

Eligible participants were veterinarians directly involved in animal welfare and animal cruelty investigations, whereas veterinarians not engaged in these duties were excluded. A total of 18 veterinarians from the DLD participated in the study. Guest *et al*. [14] suggested that a sample size of 12–20 participants is generally sufficient to achieve data saturation in qualitative research.

**Figure 2:**
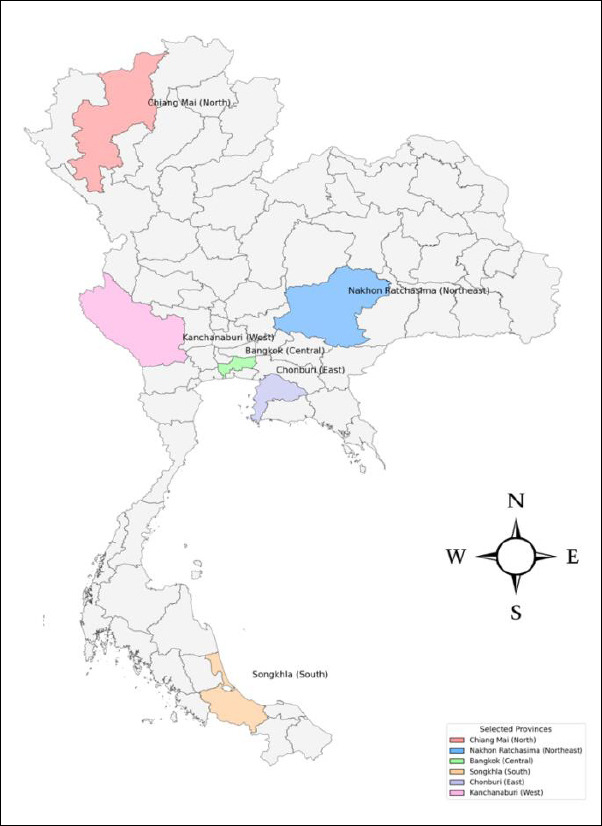
Geographic distribution of selected provinces in Thailand represented by veterinarians from the Department of Livestock Development. Highlighted provinces indicate participant representation from six regions: Chiang Mai (North), Nakhon Ratchasima (Northeast), Bangkok (Central), Chonburi (East), Kanchanaburi (West), and Songkhla (South).

Participants were purposively selected to represent all six regions of Thailand, with three veterinarians recruited from each region. This stratified purposive sampling approach ensured geographic diversity and representation of varying professional experiences while maintaining feasibility for in-depth, quote-rich qualitative analysis. The regional stratification was designed to capture perspectives from both urban and rural enforcement settings. The participating provinces included Chiang Mai (North), Nakhon Ratchasima (Northeast), Bangkok (Central), Songkhla (South), Chonburi (East), and Kanchanaburi (West) (Figure 2).

The sample size was considered adequate based on the information power concept, given the focused study aim and the use of detailed open-ended responses organized within a conceptual framework encompassing animal welfare, forensic applications, and legal dimensions.

### Data collection and analysis

This cross-sectional study was conducted between August 1 and December 31, 2024. Validated questionnaires were distributed to participants via an online link shared through digital platforms, including Facebook and Line, using Google Forms. Completed responses were compiled into a Word document for analysis.

**Figure 3:**
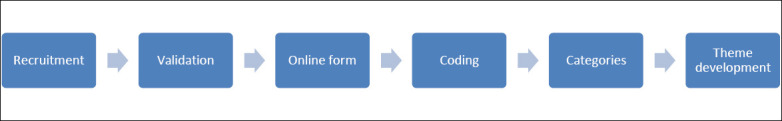
Flowchart illustrating the progression from initial coding to final thematic consolidation.

Data were analyzed using a content analysis approach. Responses were read repeatedly to generate initial codes, which were subsequently grouped into categories and overarching themes, resulting in the development of a coding tree (Figure 3). For example, the raw statement “Owners do not have enough money to provide a good quality of life for animals” was coded as “Lack of economic resources to care for animals,” categorized under “Financial limitations that impact animal welfare and quality of life,” and ultimately incorporated into the theme “Economic and social constraints affecting animal welfare.”

To minimize analytical bias, a double-blind analysis was conducted in collaboration with two additional researchers. Coders underwent calibration prior to analysis to ensure consistency in code application, and discrepancies were resolved through discussion until consensus was reached. All content analyses were performed using NVivo version 14 (https://lumivero.com/products/nvivo/). Member checking was not conducted due to the open-ended nature of the questionnaire and logistical constraints associated with recontacting all participants.

Trustworthiness of the qualitative findings was ensured in accordance with Lincoln and Guba’s criteria through double-blind coding, triangulation, maintenance of audit trails, coder reflexivity, consensus meetings, and the use of illustrative quotations [15]. The study was conducted in line with the Consolidated Criteria for Reporting Qualitative Research (COREQ) guidelines [16] (Table 1).

**Table 1:** Consolidated criteria for reporting qualitative studies (COREQ): A 32-item checklist.

Number	Guide Description	Detail
**Domain I: Research team and reflexivity**		
**Personal characteristics**		
1	Interviewer/facilitator: Which author(s) conducted the interview or focus group?	AL, YS, and NK
2	Credentials: What were the credentials of the researcher?	AL (DVM, PhD), YS (PhD), NK (PhD)
3	Occupation: What was the occupation at the time of the study?	Clinician, researcher, instructor, and forensic scientist
4	Gender: Was the researcher male or female?	Three males
5	Experience and training: What experience or training did the researcher have?	5–10 years of research experience, including qualitative data collection
**Relationship with the participants**		
6	Was a relationship established before study commencement?	No
7	Participant knowledge of the interviewer: What did the participants know about the researcher?	Participants knew that the researcher was a veterinarian and were informed of the study purpose
8	Interviewer characteristics: What characteristics were reported about the interviewer/facilitator?	Veterinarian and forensic scientist

**Domain II: Study design**		
**Theoretical framework**		
9	Orientation and theory: What is the methodological orientation that underpins the study?	Informed by animal welfare and cruelty prevention frameworks and veterinary forensic science principles
**Participant selection**		
10	Sampling: How were the participants selected?	Stratified purposive sampling with open-ended responses
11	Method of approach: How were the participants approached?	Online link via Facebook and Line
12	Sample size: How many participants were included in the study?	n = 18 participants
13	Non-participation: How many people refused or dropped out? Reasons?	None
**Setting**		
14	Setting of data collection: Where were the data collected?	Online using Google Forms
15	Presence of non-participants: Was anyone other than the participants and researchers present?	No
16	Sample description: Important characteristics of the sample?	Yes, as described in Table 2
**Data collection**		
17	Interview guide: Were the questions, prompts, and guides provided by the authors? Was it pilot tested?	Yes
18	Repeat interviews: Were repeat interviews conducted? If yes, how many?	No
19	Audio/visual recording: Did the researchers use audio or visual recording?	No
20	Field notes: Were field notes made during and after the interview or focus group?	Yes, after reviewing open-ended responses
21	Duration: What was the duration of the interviews or focus group?	Data collection from August 1 to December 31, 2024
22	Data saturation: Was data saturation discussed?	Not applicable
23	Transcripts returned: Were transcripts returned to participants for comment and correction?	Member checking not performed; trustworthiness ensured via coding and triangulation

**Domain III: Analysis and findings**		
24	How many data coders coded the data?	2
25	Coding tree: Did the authors provide a description?	Yes
26	Themes: Identified in advance or derived from data	Data-driven (inductive) themes
27	Software: What software was used to manage the data?	NVivo
28	Participant checking: Did participants provide feedback on the findings?	No
**Reporting**		
29	Quotations: Were participant quotations presented and identified (e.g., participant number)?	Yes
30	Data and findings: Is there consistency between the data presented and the findings?	Yes
31	Clarity of major themes: Were major themes clearly presented?	Yes
32	Clarity of minor themes: Were diverse cases or minor themes discussed?	Yes

AL = Athip Lorsirigool, YS = Yuttana Sudjaroen, NK = Narong Kulnides, n = number of participants.

## RESULTS

### Participant characteristics

A total of 18 veterinarians (9 males and 9 females) from the DLD responded to the questionnaire. Most respondents (n = 14) were aged 30–35 years (77.8%), followed by those aged 35–40 years (n = 2; 11.1%) and 40–45 years (n = 2; 11.1%). Regarding length of employment, 8 respondents had more than 9 years of service (44.4%), 6 had worked for 7–9 years (33.3%), 3 for 3–5 years (16.7%), and 1 for 5–7 years (5.6%) (Table 2).

**Table 2:** Demographic characteristics of veterinarians responding to the DLD.

Region	Province	Gender	Age (range, years)	Years of service in the DLD (range, years)	Code (Vet no.)
North	Chiang Mai	Male	30–35	7–9	1
North	Chiang Mai	Female	35–40	>9	2
North	Chiang Mai	Female	35–40	>9	3
Northeast	Nakhon Ratchasima	Male	30–35	7–9	4
Northeast	Nakhon Ratchasima	Male	30–35	7–9	5
Northeast	Nakhon Ratchasima	Female	30–35	>9	6
Central	Bangkok	Male	30–35	3–5	7
Central	Bangkok	Male	30–35	>9	8
Central	Bangkok	Female	30–35	7–9	9
South	Songkhla	Male	30–35	>9	10
South	Songkhla	Male	30–35	3–5	11
South	Songkhla	Female	40–45	>9	12
East	Chonburi	Male	30–35	3–5	13
East	Chonburi	Female	30–35	7–9	14
East	Chonburi	Female	40–45	>9	15
West	Kanchanaburi	Male	30–35	>9	16
West	Kanchanaburi	Female	30–35	5–7	17
West	Kanchanaburi	Female	30–35	7–9	18

DLD = Department of Livestock Development, age and years of service are presented as ranges, Vet no. indicates anonymized participant code.

### Opinions on pet abuse and inadequate animal welfare

**Figure 4:**
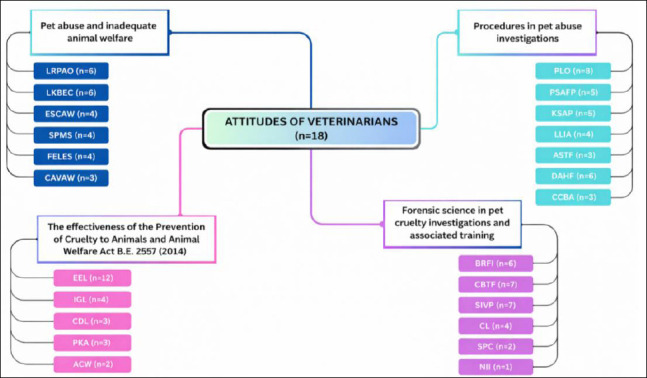
Overview of themes derived from veterinarians’ perspectives on animal welfare, legislation, and the application of forensic methods in animal cruelty investigations. Pet abuse and inadequate welfare were primarily attributed to owners’ irresponsibility and insufficient knowledge of pets’ basic needs (LRPAO, LKBEC). Veterinarians generally perceived that the Prevention of Cruelty to Animals and Animal Welfare Act B.E. 2557 (2014) contributes to reducing abuse by establishing legal accountability (EEL). However, the limited application of forensic science, due to insufficient training and the absence of standardized guidelines (PLO), highlights the need for capacity building (CBTF) and greater integration of forensic methods into routine veterinary practice (SIVP).

Six themes were identified from 25 categories and 35 coded key statements derived from veterinarians’ perspectives on pet abuse and inadequate animal welfare (Figure 4). These themes included lack of responsibility and preparation among animal owners (LRPAO), lack of knowledge, understanding, and basic ethics in animal care (LKBEC), economic and social constraints affecting animal welfare (ESCAW), structural problems related to the absence of a stray animal management system and inadequate control of animal owners (SPMS), failure of legal and enforcement systems (FELES), and cultural attitudes and values that are not conducive to animal welfare (CAVAW) (Table 3).

**Table 3:** Themes from the opinions of veterinarians on pet abuse and inadequate animal welfare.

Theme	Category	Key statement	Vet No.
LRPAO (n = 6; 33.33%)	Owner’s responsibility	Lack of awareness among pet owners	4
	Lack of responsibility	Lack of preparation among pet owners	6
		Lack of responsibility of animal owners	7, 9
	Lack of mental readiness or maturity Lack of preparation	Avoiding responsibilities and shifting the burden to others	7
		Diminished motivation to care for animals	7
		Owners’ readiness before adopting animals	9
	Lack of skills, knowledge, or resources	Animal owners are responsible	12
		Insufficient preparation for proper pet care	14

LKBEC (n = 6; 33.33%)	Awareness and knowledge of animal welfare	Lack of knowledge and understanding of the principles of animal welfare	5
	Lack of awareness and ethical responsibility	Lack of awareness of animal life	10
		Habit of using violence without remorse	11
	Lack of understanding of animal rights and animal cruelty	Lack of knowledge regarding animal protection laws	13
	Misconceptions about animal cruelty	Limited interpretation of abuse, only considering physical harm	16
	Attitudes that devalue animals	Attitudes that treat animals as objects	15
		Attitudes that fail to value animal life	15
ESCAW (n = 4; 22.22%)	Economic and societal influences	Socioeconomic conditions	1
		Economic problems affecting animal husbandry	15
	Financial limitations affecting animal welfare and quality of life	Lack of economic resources to care for animals	17
	Social factors contributing to animal cruelty	Social violence manifests in animal behaviors	15
		Violence and lack of morality in society	16

SPMS (n = 4; 22.22%)	Deficiencies in the management of stray animals	Stray animals without owners to care for them	2
		Animals without owners or with unclear ownership	3
		Insufficient control of stray animal populations	8
	Animal control regulations and enforcement	Insufficient control of animal husbandry and population	1
	Negative community attitudes toward stray animals	Community views animals as a problem	3
	Abusive responses to stray animal issues	Aggressive responses directed at the animals	3
FELES (n = 4; 22.22%)	Failure of the justice system to protect animals	Law enforcement lacks seriousness	11
	Deficiencies in the enforcement of animal law	Strict policies or control measures	18
		Law enforcement does not take the issue seriously	15
	Weak deterrence through legal penalties	Continue living without facing legal punishment	11
	Deficiencies in the monitoring and reporting system	Lack of reports or witness testimony	11
	Lack of legal awareness and understanding of animal rights	Lack of knowledge regarding pet protection laws	13

CAVAW (n = 3; 16.67%)	Conflict between local culture and universal values	Consumption of pets	15
	Human attitudes and ethics toward animals	Insufficient compassion and empathy toward animals	18
	Behaviors and attitudes of pet owners	Negligent behavior in the care of animals	1

LRPAO = Lack of responsibility and preparation by animal owners, LKBEC = Lack of knowledge, understanding, and basic ethics of animal care, ESCAW = Economic and social constraints affecting animal welfare, SPMS = Structural problems related to the lack of a stray animal management system and inadequate control of animal owners, FELES = Failure of the legal and enforcement systems, CAVAW = Cultural attitudes and values that are not conducive to the welfare of animals, n = number of veterinarians expressing the theme, percentages are based on the total number of respondents (n = 18), Vet No. indicates anonymized participant code.

### LRPAO

This theme comprised five categories, eight key coded statements, and responses from six veterinarians. Respondents highlighted owners’ inability to properly care for pets, irresponsible abandonment, and insufficient mental readiness, maturity, skills, and knowledge required for appropriate animal care. One respondent noted, “Most of the causes arise from pet owners who, after a certain period of time, become bored and no longer want to keep their animals” (Veterinarian 7, Central). Another stated, “The owner was unable to provide adequate pet care” (Veterinarian 14, East).

### LKBEC

This theme included five categories, seven key coded statements, and input from six veterinarians. Respondents reported that owners often lack knowledge of animal welfare principles, are unaware of their ethical responsibilities, have a limited understanding of animal rights and abusive behaviors, misunderstand what constitutes animal cruelty, and hold attitudes that devalue animals. As one veterinarian explained, “This is caused by owners’ lack of understanding of animal welfare principles, how to appropriately manage animals, and what actions are considered violations of those principles” (Veterinarian 5, Northeast). Another noted that “Knowledge and understanding of the term ‘animal cruelty’ remain limited” (Veterinarian 16, West).

### ESCAW

This theme consisted of three categories, five key coded statements, and responses from four veterinarians. Economic hardship and social factors were perceived to directly affect animal welfare and quality of life. Social stress and violence were also reported to influence animal cruelty behaviors. One respondent stated, “Owners do not have enough money to provide a good quality of life for animals” (Veterinarian 17, West), while another emphasized that “Poverty can make it impossible for individuals to properly care for their animals” (Veterinarian 15, East).

### SPMS

This theme comprised four categories, six key coded statements, and responses from four veterinarians. Identified issues included the absence of a systematic stray animal management program, lack of animal control regulations, absence of owner identification systems, negative community attitudes toward stray animals, and structural weaknesses that lead to cruel responses. One veterinarian commented, “No control of raising, no control of the animal population” (Veterinarian 1, North), while another stated, “Stray dogs lack an effective population control system” (Veterinarian 8, Central).

### FELES

This theme included five categories, six key coded statements, and responses from four veterinarians. Respondents described failures in the justice system to adequately protect animals, insufficient punishment mechanisms, lack of deterrence, gaps in monitoring and reporting, and limited public understanding of animal rights and legal penalties. Two respondents remarked, “Laws are not comprehensive, punishments are not severe, enforcement is not strict, and offenders can continue to live without legal punishment” (Veterinarians 11 and 15, South and East).

### CAVAW

This theme comprised three categories, three key coded statements, and responses from three veterinarians. Cultural conflicts between local practices and universal welfare values, as well as human attitudes and ethical traits, were perceived to influence animal abuse. Two respondents noted, “Human characteristics that lack compassion for animals and view them as objects” (Veterinarians 15 and 18, East and West).

### Opinions on the effectiveness of animal welfare legislation

Responses concerning the Prevention of Cruelty to Animals and Animal Welfare Act B.E. 2557 (2014) were grouped into five themes based on 12 categories and 32 coded key statements: effectiveness and enforcement of the law (EEL), improvements and gaps in the law (IGL), clarity and definitions within the law (CDL), public communication, knowledge, and awareness (PKA), and animal control and welfare (ACW) (Table 4).

**Table 4:** Themes from veterinarians’ opinions on the effectiveness of the Prevention of Cruelty to Animals and Animal Welfare Act B.E. 2557 (2014) in addressing pet abuse

Theme	Category	Key statement	Vet No.
EEL (n = 12; 70.59%)	Effectiveness and impact of the law	The impact of the law remains limited	1
		The law provides clear penalties that deter wrongdoing	4
		Laws play a key role in reducing animal cruelty	8
		Laws can help reduce some of these issues	14, 15
		To some extent, laws have helped reduce	18
		abuse	
		The law is helpful to some extent but does not need to be expanded	10
		The law is somewhat effective and does not need to be amended	12
		Laws impact only those who have a conscience	13
	Role of law in animal protection	Laws contribute positively to animal protection	15
		Laws contribute to raising awareness and reducing violence against animals	16
	Law enforcement and penalties	Enforcement is not yet widespread or effective	15
		Strict law enforcement is necessary	18
		The relevant agencies do not play a role in enforcement	7
		Stricter inspections and harsher penalties should be implemented	17
IGL (n = 4; 23.53%)	Strengthening legal penalties	The penalty is insufficiently severe	11
		The length of prison terms and the fine amount should be increased	11
	Legal and systemic gaps in the management of stray animals	The law provides little or no protection for homeless animals	3
		The animal adoption system remains ineffective	6
	Legal coverage of protected animals	Unclear regulations regarding which animals are prohibited from being killed for consumption	6
		Information about the specific animal group is not sufficiently detailed	15
CDL (n = 3; 17.65%)	Clarity and ambiguity of legal definitions	The definition of “animal owner” remains unclear	6
		The definition of animal cruelty is ambiguous	15
		The classification of animal abandonment as cruelty remains unclear	16
PKA (n = 3; 17.65%)	Promotion of education and awareness	Laws contribute to increasing awareness of animal welfare	5
		Many people still do not understand the law	15
		The public should be informed about the definition of animal cruelty	16
	Communication and accessibility of the law	Insufficiency of mechanisms for disseminating knowledge to the public	15
	Community participation	Participation of the public plays a crucial role in enforcement	15
ACW (n = 2; 11.76%)	Animal care and management	Pet owners should have clearly defined responsibilities	9
		Not treating sick animals could be deemed animal cruelty	16
		Whether keeping numerous animals in cages constitutes cruelty should be addressed	16
	Nutrition and feeding of animals	Feeding animals with harmful or inappropriate food should be classified as cruelty	16

*Vet No. 2 did not answer the questions. EEL = Effectiveness and law enforcement, IGL = Improvements and gaps in the law, CDL = Clarity and definitions of the law, PKA = Public communication, knowledge, and awareness, ACW = Control and welfare of animals, n = number of veterinarians expressing the theme, percentages are based on the total number of respondents (n = 17), Vet No. indicates anonymized participant code, Vet No. 2 did not answer the questions.

### EEL

This theme comprised three categories, 14 key coded statements, and responses from 12 veterinarians. Respondents acknowledged that clear legal penalties help deter wrongdoing, raise awareness, and reduce violence against animals, although enforcement remains inconsistent. One respondent stated, “It can help reduce the problem to a certain extent, but there are still limitations. Law enforcement is not widespread” (Veterinarian 15, East). Another added, “The law can help, but acts of abuse are still occasionally observed” (Veterinarian 17, West).

### IGL

This theme included three categories, six key coded statements, and responses from four veterinarians. Respondents emphasized the need for stronger penalties, clearer definitions of protected animals, and better legal coverage for stray animals. As noted, “Penalties should be more severe. Problems will persist if animal husbandry is not systematically managed” (Veterinarians 3 and 6, North and Northeast).

### CDL

This theme comprised one category, three key coded statements, and responses from three veterinarians. Respondents highlighted ambiguity in the definitions of animal cruelty and “animal owner.” One veterinarian explained, “The definition of ‘animal owner’ may cause issues regarding people who are not owners but provide food to stray animals” (Veterinarian 6, Northeast). Another noted uncertainty regarding whether animal abandonment constitutes cruelty (Veterinarian 16, West).

### PKA

This theme included three categories, five key coded statements, and responses from three veterinarians. Respondents emphasized the importance of educating animal owners and improving public understanding of animal cruelty. One stated, “This will help animal owners understand which actions are appropriate and which are wrong or contrary to animal welfare principles” (Veterinarian 5, Northeast).

### ACW

This theme comprised two categories, four key coded statements, and responses from two veterinarians. Respondents stressed the need to regulate animal ownership, health care, living environments, and nutrition. One veterinarian stated that it should be clarified whether keeping large numbers of animals in cages or failing to treat sick animals constitutes cruelty (Veterinarian 16, West).

### Opinions on procedures in pet abuse investigations

Veterinarians’ responses regarding investigative procedures were categorized into seven themes derived from 26 categories and 52 key coded statements: practical limitations and obstacles (PLO), procedures and standards of animal forensic science practice (PSAFP), knowledge, skills, and availability of personnel (KSAP), laws and limitations on inspection authority (LLIA), application of science and technology in forensics (ASTF), differences between animal and human forensic processes (DAHF), and coordination and collaboration between agencies (CCBA) (Table 5).

**Table 5:** Themes from veterinarians’ opinions on procedures in pet abuse investigations.

Theme	Category	Key statement	Vet No.
PLO (n = 8; 47.06%)	Gaps and limitations in the investigative process	Lack of proper verification procedures	2
		Delays in field visits or evidence loss	2
		Investigate upon notification	5
		Insufficient in-depth investigation	7
		Examining the external conditions of animals and their surroundings	6
	Limitations of standards and guidelines	The verification process is less comprehensive than that for humans	4
		Following the prescribed procedures	14
	Lack of adequate operational resources	Budgetary restrictions	4
	Lack of forensic application in investigations	More emphasis is placed on interviews than on the collection of physical evidence	7
		Forensic procedures are not used	13
	Collecting information in investigations	Use of investigative methods focusing on individuals	11
		Investigations are mainly conducted through questioning	13
	Satisfaction with agency processes	The applied procedures are appropriate to the context	14
PSAFP (n = 5; 29.41%)	Audit and inspection procedures	Visit the scene to assess the incident	5
		Fieldwork and on-site responsibilities	15
	Data collection and verification	Conducting an inquiry to determine the cause of death	5

		Conduct an on-site incident inspection	9
	Notification setup guide	The victim has been informed of the incident	15
	Veterinary forensic reporting	Analysis, conclusions, and preparation of reports	15
	Standardization in forensic audit	Procedures in each agency differ	15
		Inspection standards similar to those used in human cases	16
		Lack of systematic animal procedures	17
KSAP (n = 5; 29.41%)	Skills and readiness gaps in forensic personnel	Lack of training in animal forensic science	6
		This task requires veterinary expertise	15
		Lack of training and standardized guidelines for veterinarians	17
	Knowledge and awareness gap	Limited knowledge of human forensic science procedures	10
		Veterinarians should be trained in human forensic science principles	8
		The actual implementation does not meet the standard	8
LLIA (n = 4; 23.53%)	Legal role and enforcement	Adherence to principles of law in operations	6
		Legal action taken upon a guilty verdict	15
	Limitations and barriers to enforcement	Entry into the area requires notification to the owner	6
		Access limitations to internal premises	10
	Legal frameworks and regulations	Compliance with procedures established under specific legal provisions	12
		Animal-specific laws and regulations	15
ASTF (n = 3; 17.65%)	Use of technology and visual evidence	Considering the use of photographic and video evidence	11
		Photographs and videos should be recorded to provide evidence	15
	Physical and scientific evidence use	Ensure proper collection of physical evidence	15
		Support for scientific testing is essential	16
		Sample collection for laboratory testing	15
	Analysis of evidence and judicial process	Conducting analysis to identify perpetrators or determine causes	15
	Forensic veterinary procedures and necropsy	Necropsy of animal remains to verify the cause of death	16
		Necropsy of animal remains to determine the cause of death	15
Differences between animals and humans in forensic science processes (n = 6; 35.29%)	Differences between animal and human forensic studies	The verification process differs from that of humans	1
		The process differs from that of humans	4
	Differences between animal and human forensic processes	Procedures that differ from those used for humans	7
		Animal forensic processes differ from those used in human cases	12
	Differences in testing between humans and animals	The process for investigating animal incidents is not as clear as that for humans	17
	Similarities between animal and human forensic processes	Some steps are similar to those used in human forensics	18
CCBA (n = 3; 17.65%)	Multiagency collaboration and coordination	A joint inspection should be coordinated with relevant agencies	6, 10
		Joint inspection of the area was conducted	6, 10
		Coordinate efforts with local agencies	10
	Challenges related to coordination and field operations	Public cooperation in animal-related cases is lower than in human cases	18

*Vet No. 3 did not answer the questions. PLO = Practical limitations and obstacles, PSAFP = Procedures and standards of practice in animal forensic science, KSAP = Knowledge, skills, and personnel availability, LLIA = Laws and limitations on inspection authority, ASTF = Application of science and technology in forensics, CCBA = Coordination and collaboration among agencies, n = number of veterinarians expressing the theme, percentages are based on the total number of respondents (n = 17), Vet No. indicates anonymized participant code, Vet No. 3 did not answer the questions.

### PLO

This theme included six categories, 13 key coded statements, and responses from eight veterinarians. Respondents described gaps in investigative rigor, reliance on non-forensic methods, delayed responses, limited resources, and minimal application of forensic techniques. Two respondents stated, “There is no real inspection system. It is merely an inquiry, not using forensic methods to support the process” (Veterinarians 2 and 13, North and East).

### PSAFP

This theme comprised five categories, nine key coded statements, and responses from five veterinarians. Respondents reported a lack of standardized procedures for animal abuse investigations and emphasized the need for manuals aligned with human forensic standards. Two respondents noted, “Different local agencies have different investigation procedures. They should have the same standards of inspection as humans” (Veterinarians 15 and 16, East and West).

### KSAP

This theme included two categories, six key coded statements, and responses from five veterinarians. Respondents highlighted insufficient training and limited readiness among personnel. One stated, “In veterinary medicine, only a few have been trained in inspecting places” (Veterinarian 17, West).

### LLIA

This theme comprised three categories, six key coded statements, and responses from four veterinarians. Legal restrictions on accessing private property were identified as a major obstacle. As noted, “Without the permission of the property owner, only the surrounding area can be inspected” (Veterinarian 10, South).

### ASTF

This theme included four categories, eight key coded statements, and responses from three veterinarians. Respondents encouraged greater use of forensic examinations, laboratory methods, and photographic or video evidence to strengthen judicial outcomes.

### DAHF

This theme comprised four categories, six key coded statements, and responses from six veterinarians. Respondents noted that although some procedures are similar, animal forensic processes are less developed and less standardized than those used in human cases.

### CCBA

This theme included two categories, four key coded statements, and responses from three veterinarians. Respondents emphasized the need for multiagency collaboration and noted that public cooperation in animal cases is often lower than in human cases.

### Opinions on the use of FS in pet cruelty investigations and training

Six themes were identified from 20 categories and 43 key coded statements: benefits and roles of forensic science in animal investigations (BRFI), capacity building and training in forensic science (CBTF), support and integration of forensic science in veterinary practice (SIVP), national impact and image (NII), challenges and limitations (CL), and standards, processes, and collaboration (SPC) (Table 6).

**Table 6:** Themes related to the use of FS in pet cruelty investigations and associated training.

Theme	Category	Key statement	Vet No.
BRFI (n = 6; 33.33%)	Benefits and roles of forensic science	The application of FS principles is beneficial to relevant agencies	3
		Recognizing the benefits of forensic science in animal studies	4
		Forensic science plays a supportive role in the management of operations	5
	Use and reliability of scientific evidence	Forensic science assists in evidence discovery	6
		Wound analysis can be used to demonstrate wrongdoing	15
		Assist in identifying perpetrators through scientific evidence	15
		Use of forensic science to provide evidence	5
		Evidence collection methods have become more accurate	15
	Development and efficiency of the justice system	Forensic science enhances investigation efficiency	15
		Enhancing the accuracy and fairness of prosecuting perpetrators	15
		Exploring new perspectives on the use of technology and evidence	15
		Accelerating the litigation process	15
		The application of forensic science principles leads to more structured investigations	18
CBTF (n = 7; 38.89%)	Human resource development and training	Training should be provided to improve work performance	5
		Provide support for professional training	6
		Recognize the importance of training organization	15
		Support training in the application of FS principles	17
	Advancement of veterinary knowledge and skills	Training programs should be organized to develop forensic veterinarians’ potential	16
		Training should be organized to increase forensic science knowledge	12
		Training should be provided to enhance forensic science knowledge	14
	Training programs and workshops	Workshops and field training are recommended	15
		The curriculum should cover all processes	15
SIVP (n = 7; 38.89%)	Promotion and integration of FS	Agree with the use of forensic methods	1
		Supporting the application of principles of forensic science	2
		There is clear agreement on the use of FS principles	8
		Forensic methods are useful in case investigations	17
		A lack of scientific evidence renders prosecution impossible	16
	Supporting justice and the legal process	Training enhances the effectiveness of identifying offenders	11
		The use of forensic science principles helps generate legal evidence for prosecuting perpetrators	9
	Role of government agencies in animal cruelty cases	Confirmation of the incident by authorities makes the case more credible	16
CL (n = 4; 22.22%)	Obstacles to the integration of forensic science	The application of forensic science to animals remains unclear	4
	Operational limitations owing to legal restrictions	Field visits require the presence of police to avoid violating privacy laws	10
	Operational obstacles due to a lack of human resources	A small staff size can make the use of forensic methods burdensome	13
	Limitations from social values and attitudes	Traditional social values regarding animals pose challenges to the development of innovative approaches	7
SPC (n = 2; 11.11%)	Standardization and quality assurance	Promoting practice consistency	17
		Developing a standardized inspection protocol	17
	Reliability and validity of investigations	Having standard reference data makes the process more reliable	18
	Collaboration and partnerships	Using scientific methods increases cooperation among stakeholders	18
	Use of data in investigations	Necropsy results can be stored in a database as a reference for determining the cause of infection	18
NII (n = 1; 5.56%)	National-level advancement of animal welfare	Thailand should be aware of the application of forensic science principles in veterinary medicine	15
		Promote effective and standardized law enforcement	15
	Impact of knowledge development	Training represents a valuable long-term investment in the field	15
	National image	Enhancing Thailand’s image and credibility in animal welfare	15

FS = forensic science, BRFI = Benefits and roles of forensic science in animal investigations, CBTF = Capacity building and training in forensic science, SIVP = Support and integration of forensic science in veterinary practice, CL = Challenges and limitations, SPC = Standards, processes, and collaboration, NII = National impact and image, n = number of veterinarians expressing the theme, percentages are based on the total number of respondents (n = 18), Vet No. indicates anonymized participant code.

### BRFI

Respondents emphasized that forensic science enhances investigative accuracy, evidentiary credibility, and judicial efficiency. One veterinarian stated that forensic techniques “enhance both the accuracy and effectiveness of evidence collection and case resolution” (Veterinarian 15, East).

### CBTF

Respondents highlighted the need for workshops and structured training curricula to develop veterinary forensic capacity. As two respondents noted, “If there is training, it will be very useful in the work” (Veterinarians 5 and 6, Northeast).

### SIVP

Respondents expressed strong support for integrating forensic science into animal cruelty investigations, emphasizing improved evidence quality and investigative effectiveness.

### NII

Some respondents noted that applying forensic science could strengthen Thailand’s animal welfare system and enhance national credibility in veterinary practice.

### CL

Respondents identified shortages of trained personnel, legal access barriers, and prevailing social values as key challenges to implementing forensic science in veterinary contexts.

### SPC

Respondents emphasized that forensic science could support standardized procedures, improve consistency in inspections, and enhance intersectoral collaboration, thereby increasing the credibility of investigative outcomes.

## DISCUSSION

### Drivers of pet abuse and inadequate animal welfare

In this study, veterinarians reported that pet abuse and inadequate pet welfare management were primarily driven by owners’ irresponsibility in providing appropriate care and by insufficient knowledge and understanding of pets’ basic needs (LRPAO and LKBEC; 33.33%). These findings are consistent with the work of Sarıal and Bozkurt [17], who examined animal welfare perspectives among animal owners in Turkey and reported that welfare outcomes are closely linked to the type of animal kept, the provision of nutritionally adequate food, and the animal’s health status. That study also highlighted the important role of nongovernmental organizations in improving animal welfare.

Socioeconomic factors were also identified as major contributors to compromised pet welfare (22.22%). Financial constraints can limit owners’ ability to provide adequate veterinary care, nutrition, and appropriate living conditions. Similarly, *McDowall et al*. [18] demonstrated that economic factors strongly influence the physical, environmental, and developmental health of pets, as animals require consistent veterinary care, balanced nutrition, and timely medical treatment when ill. In economically constrained settings, pets may have limited access to vaccinations, and euthanasia may be considered due to the high cost of treating certain viral infections, such as parvovirus. From a social perspective, pets living in supportive environments with attentive owners and positive human–animal interactions tend to exhibit better mental health outcomes. In contrast, exposure to violent environments, including households affected by domestic violence, has been associated with an increased risk of pet abuse [19].

Structural challenges in stray pet management and household pet population control also emerged as significant concerns (22.22%). The absence of coordinated, long-term systems for registration, sterilization, and population control has reduced authorities’ capacity to prevent uncontrolled breeding and the continued growth of stray animal populations across multiple provinces. In some areas, stray animals are subjected to violent handling, raising serious animal cruelty concerns. In addition, guidelines addressing owners’ readiness to care for pets, including limits on the number of animals per household and requirements for microchipping, remain unclear. As a result, regulations and penalties are often ineffective, largely due to difficulties in enforcing pet inspections (FELES; 22.22%). A previous study has suggested that penalties for animal cruelty are frequently perceived as lenient, contributing to repeat offenses and prompting recommendations for increased maximum penalties and more consistent judicial application [20]. Cultural factors further influence behaviors related to pet abuse (CAVAW; 16.67%). In some regions, pet consumption persists under the belief that it is not unlawful, reflecting limited public awareness of existing legal prohibitions. Comprehensive public awareness campaigns and nationwide surveys are therefore needed to clarify which animals may be legally consumed and which are protected, particularly given the severe legal penalties associated with such practices.

### Effectiveness and limitations of animal welfare legislation

With respect to the Prevention of Cruelty to Animals and Animal Welfare Act B.E. 2557 (2014), most participating veterinarians agreed that the Act contributes to reducing pet abuse by establishing legal accountability for offenders (EEL; 70.59%). Nevertheless, respondents emphasized the need for further legal development and the closure of existing loopholes (IGL; 23.53%), particularly regarding the treatment of stray animals, animal abandonment, and the killing of animals for consumption, all of which remain legally and ethically contested.

Establishing clear legal definitions of “pet ownership,” “pet abandonment,” and “pet cruelty” within the Act was considered essential for improving enforcement and strengthening animal welfare protection (CDL; 17.65%). Although these terms are defined in the legislation, limited public understanding underscores the need for improved communication and broader awareness initiatives. *Caldwell et al*. [21], in a retrospective analysis of animal cruelty cases in New York City, reported that most cases involved dogs and cats, with dog injuries more frequently resulting from neglect and cat injuries more often associated with nonaccidental harm. These findings highlight the importance of identifying pet ownership and assessing whether observed injuries are consistent with abuse. Accordingly, public awareness campaigns and community outreach programs should be implemented to improve understanding of animal cruelty and enhance the effective application of the law (PKA; 17.65%). In addition, Striwing and Sarenbo [22] reported that neglecting a dog to the extent that it causes injury to another person constitutes criminal negligence, emphasizing the responsibility of owners to maintain appropriate control when adopting pets. Consistent with these findings, veterinarians in the present study supported legal limits on the number of pets per household and requirements for owners to provide care that meets animals’ basic needs (ACW; 11.76%).

### Role of forensic science in pet abuse investigations

Internationally, forensic methods are increasingly used to investigate pet abuse cases, enabling determination of the cause of injury or death and assisting in perpetrator identification [11, 23]. In Thailand, however, forensic science has not yet been widely integrated into pet abuse investigations due to limited training opportunities and the absence of standardized guidelines (PLO; 47.06%). In contrast, reports from other Asian countries, including Taiwan and Japan, indicate that forensic science has been incorporated into veterinary practice, encompassing wound pattern analysis, DNA extraction from wounds to determine injury causation, and necropsies in cases of death associated with inadequate welfare management [24, 25].

Veterinarians in this study noted that investigative procedures for animals remain unclear, despite some similarities with human forensic processes (DAHF; 35.29%). Consequently, the development of standardized forensic practice guidelines (PSAFP; 29.41%) and targeted training for veterinarians involved in examining abused pets (KSAP; 29.41%) were identified as priorities. These findings align with those of *Mores et al*. [12], who emphasized that forensic examinations must follow systematic procedures consistent with chain-of-evidence principles. The adoption of scientific technologies analogous to those used in human forensics can improve evidentiary accuracy and increase judicial confidence (ASTF; 17.65%). For instance, in cases of pet abandonment, detecting owner DNA on animals can assist in identifying and tracing responsible individuals [26]. Nevertheless, fundamental practices, including high-quality photographic or video documentation and comprehensive medical or necropsy reports, remain essential. Effective implementation of forensic science in pet abuse investigations also requires close interagency coordination (CCBA; 17.65%), as legal restrictions on accessing private property necessitate collaboration among police officers, community leaders, and veterinarians (LLIA; 23.53%).

### Implications for veterinary practice and future research

At present, investigations of pet abuse in Thailand have not explicitly incorporated forensic science. Participating veterinarians recommended its broader application to enhance evidentiary reliability and investigative consistency (BRFI; 33.33%). However, limitations remain, including shortages of trained forensic specialists, insufficient personnel, and constrained funding (CBTF and CL; 38.89% and 22.22%, respectively). Increasing public awareness of the role of forensic science in animal investigations is also necessary (SIVP; 38.89%), as reducing animal cruelty can strengthen national and international standing in animal welfare and cruelty prevention (NII; 5.56%).

**Figure 5:**
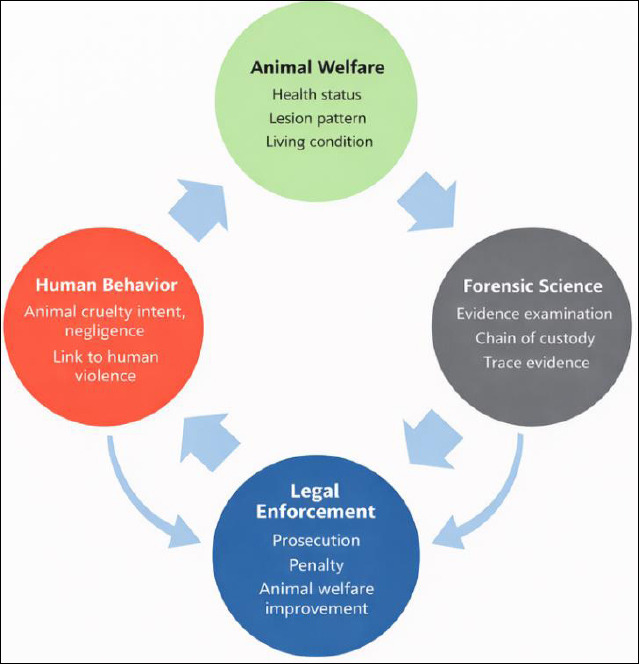
Conceptual framework depicting the interrelationships among animal welfare, human behavior, forensic science, and legal enforcement. Integrated assessment of animal welfare conditions, human behavioral factors, and forensic evidence informs legal decision-making, supports the identification of animal cruelty, and contributes to the improvement of animal welfare outcomes.

This study demonstrates that determining whether animal welfare conditions constitute cruelty requires a comprehensive assessment of animal health, living conditions, and owner or perpetrator behavior, particularly when distinguishing intentional harm from deliberate neglect. Integrating forensic methods strengthens such assessments by enhancing evidentiary credibility through trace analysis and proper chain-of-custody procedures, thereby reinforcing animal cruelty legislation and supporting appropriate penalties (Figure 5). These findings align with the One Welfare concept, which emphasizes the interconnections among animal welfare, social factors, and human well-being [3], and highlight the expanded role of veterinarians in promoting responsible ownership and strengthening law enforcement capacity.

Future research should prioritize developing comprehensive handbooks on animal cruelty, animal welfare, and relevant legislation to improve public understanding and assess pet owners’ knowledge. Additional studies are needed to evaluate whether accessible outreach programs can effectively reduce animal cruelty incidence. Practical manuals on forensic examination of abused animals, accompanied by structured training programs for veterinarians and relevant agencies, would further promote standardized investigative practices. Legal mechanisms that enable or require veterinarians to report suspected cruelty to law enforcement, as implemented in the United States [27], should also be explored.

Beyond these initiatives, future studies should adopt multi-stakeholder qualitative comparative designs incorporating perspectives from veterinarians, law enforcement officers, community organizations, and policymakers. Forensic case-audit studies are also warranted to evaluate evidence-handling quality and identify procedural gaps across agencies. Ultimately, the development of a national Veterinary Forensic Readiness Framework in Thailand would provide a structured foundation for standardized and effective animal cruelty investigations.

## CONCLUSION

This study demonstrated that pet abuse and inadequate animal welfare in Thailand are driven by multiple, interrelated factors spanning individual, structural, legal, and sociocultural domains. The most prominent contributors were owners’ lack of responsibility and preparedness and insufficient knowledge and ethical understanding of animal care (LRPAO and LKBEC). Economic and social constraints (ESCAW) further limited owners’ capacity to provide adequate care, while weaknesses in stray animal management systems (SPMS) contributed to persistent welfare challenges. Although existing legislation was perceived to play a meaningful role in reducing cruelty through legal accountability (EEL), gaps in enforcement, unclear definitions, and limited public awareness (IGL, CDL, and PKA) reduced its overall effectiveness. Importantly, veterinarians identified substantial shortcomings in investigative procedures, particularly the limited application of forensic science, lack of standardized protocols (PSAFP), and insufficient training and personnel capacity (KSAP), which collectively undermined evidence reliability and case outcomes.

The findings highlight the need for a more integrated and operational approach to addressing pet abuse and animal welfare. Strengthening owner education and public awareness initiatives is essential to address LRPAO and LKBEC, while coordinated strategies for stray animal population control are required to mitigate SPMS-related challenges. From an enforcement perspective, clearer legal definitions, consistent inspections, and proportionate penalties are needed to enhance the deterrent effect of existing laws. The study also underscores the practical value of integrating forensic science into routine investigations, as supported by veterinarians’ views on its benefits (BRFI). Developing standardized investigative protocols, expanding forensic training opportunities, and promoting interagency collaboration (CCBA) would substantially improve evidentiary quality and judicial confidence.

A key strength of this study lies in its national scope and qualitative design, which enabled in-depth exploration of frontline veterinarians’ perspectives across all regions of Thailand. The use of stratified purposive sampling ensured representation from diverse geographic and enforcement contexts, while systematic content analysis enhanced analytical rigor. By linking welfare, legal, and forensic dimensions, the study provides one of the first comprehensive empirical insights into the practical realities of animal cruelty investigations in Thailand.

Several limitations should be acknowledged. Data were collected through an online questionnaire, which may have introduced self-selection bias and limited opportunities for follow-up clarification. The study focused exclusively on veterinarians employed by the DLD, thereby excluding perspectives from other key stakeholders such as law enforcement officers, nongovernmental organizations, private practitioners, and policymakers. In addition, the absence of direct case file analysis limited the ability to objectively assess investigative outcomes or evidentiary quality. These factors may constrain the generalizability of the findings beyond similar regulatory contexts.

Future research should adopt multi-stakeholder qualitative and mixed-method designs to capture a broader range of perspectives on animal cruelty prevention and enforcement. Empirical evaluation of forensic practices through case-audit studies would be valuable for identifying procedural gaps and weaknesses in evidence handling. The development and pilot testing of standardized forensic guidelines, accompanied by structured training programs for veterinarians and related agencies, represent critical next steps. Longitudinal studies assessing the impact of public awareness campaigns and legal reforms on animal cruelty incidence would further inform policy and practice.

Overall, this study highlights that addressing pet abuse and inadequate animal welfare requires more than legislative frameworks alone. Effective prevention depends on responsible ownership, public awareness, robust enforcement mechanisms, and the systematic integration of forensic science into investigations. By strengthening investigative capacity, standardizing procedures, and fostering interagency collaboration, Thailand can enhance animal welfare protection and ensure the consistent application of animal cruelty legislation. These efforts align with the One Welfare concept and reinforce the expanding role of veterinarians in safeguarding animal well-being, supporting justice systems, and contributing to broader societal benefits.

## DATA AVAILABILITY

The supplementary data can be made available from the corresponding author upon request.

## AUTHORS’ CONTRIBUTIONS

AL, YS, and NK: Conceptualization, methodology, investigation, data curation, and formal analysis. AL: Visualization and supervision. AL and YS: Drafting and editing of the manuscript. NP, SP, AR, MI, SC, KS: Investigation, data curation, and formal analysis. All authors have read and approved the final version of the manuscript.
